# Nuggets

**Published:** 1891-05

**Authors:** C. Carleton Smith

**Affiliations:** Philadelphia


### NUGGETS.

C. CARLETON SMITH, M. D., PHILADELPHIA.

In scrofulous children, the septum narium becomes covered with scurfy, crusty deposits, sometimes spreading to the nostrils. Such a condition will call your attention to Bovista.

Bovista, also, causes such great dryness of the mouth as to cause a decided feeling of sand scattered over the buccal surface.

In children who have contracted the habit of stuttering, especially when engaged in reading, do not overlook Bovista.

The Bovista patient often complains that there is a piece of ice lodged in the pit of the stomach. Bovista is also worthy of study in severe cases of illness where we find the urine green, or yellowish-green.

Bovista will also be frequently called for in women who invariably have leucorrhœa after each menstrual period. The discharge like white of egg, and taking on a greenish color.

In some malignant cases of scald-head in scrofulous children, a cure cannot be effected without the aid of Bromine.

Women needing Bromine suffer with constant emissions of flatulence from the vagina. Lyc. has flatulence escaping from mouth of womb.

Bromine is also invaluable in many cases of dyspnœa preventing fast walking or ascending a height, all such efforts being followed quickly by complete exhaustion.

The Calcarea-carb. patient is aroused from a sound sleep every morning by a most violent aching in vertex region, lasting a longer or shorter period.

Patients with frequent snapping in head and ears as of electric sparks, will often need Calc-carb. in the course of treatment; and also individuals whose vision is too long.

Children who frequently chew and swallow in their sleep will remind you of Calc-carb. Bry., also, has a similar symptom.

White stools as if deficient in bile, streaked with blood, indicate Calc-carb.

Patients who are continually harping about being magnetized often need Calc-carb. on this account.

Forms of acute dyspepsia in which the least morsel of food swallowed causes the most violent pains are often cured by Calc-phos.

In some forms of acute lumbago, when the patient cannot make the slightest motion without screaming with the pain produced, Calc-phos. is invaluable.

Under Capsicum, we have sensation of cold water and of cold, but not of ice. Which leads the patients to ask for some hot, spicy drink to warm the cold spots up.

Cramps in the aged at night are of frequent occurrence, especially in calves of legs; for which we have Rhus-tox., Sulph., Colos-terrapina, Electro-mag., etc. But for cramps in *soles* of feet occurring nightly, think of Eugenia-iambos.

Patients who come to you complaining of taste in their mouths like rancid grease; do not give, necessarily, Pulsatilla, but sometimes study Euphorbium-off.

At the commencement of some obscure cerebral disorders, patients will raise their legs high in walking, imagining they are stepping over elevated places, Euphorbium must be studied in this connection, also, Agaricus-mus.

Euphorbium-off. has inflammation of eyelids of a very pale color, and all objects appear larger than they really are to the sight.

In asthma, when the slow and difficult breathing is greatly improved by both walking and talking, or by reading and writing steadily, think of Ferrum-aceticum. Expectoration of greenish pus comes under the proving of Ferrum-acet. And as a congener of this, think of Carbo-animalis; though the latter has pus stinking most horribly. When after a severe strain from lifting, a patient complains that he has all the sensations of an umbilical hernia occurring, Granatum will allay his fears by removing the feeling, and hence preventing what might have been a rupture.

Spasmodic action of the throat, causing constant deglutition, will often be best treated by Graphites; this same drug must also be studied in cases of women who become hoarse during each menstrual period. Profuse sweats during the menses also belong to Graphites, while it must not be forgotten in those cases of illness where the breath of the patient is laden with a strong odor of urine. In excessive ill-humor and discontent, we must never overlook Fluoric acid. Dr. Hering once stated that he gave this remedy to an old invalid lady who vehemently quarreled with nurses, relatives, and the whole house. Two doses of the drug soon brought about a most cheerful and contented spirit.

Dr. Hering also called attention to the fact that in intense itching of old *cicatrices*, even after thirty-two years' existence, this drug is the curative.

In ophthalmia, when the patient can bear with comfort the light of day, but cannot the artificial light at night, think of Graphites.

I have had patients come to my office, frequently asking for a remedy to stop fits of uncontrollable sneezing. When you meet such, give them Gummi-gutti.

The weak, debilitated feeling which overcomes many women at each menstrual period, accompained with more or less painful bearing-down, will be relieved by Hæmatoxylon.

It is well to remember the three leading remedies which have greasy pellicles floating on the urine, viz.: Hepar, Phosphorus, and Sulphur.

If in varicose limbs we find intense soreness to touch of the knotted veins, Hamamelis will give us must brilliant results. Not the tincture, nor the extract, but the potentized drug.

In the treatment of diabetes, keep in mind Nitrate of Uranium, which has this characteristic group of symptoms: Constant inclination to urinate with forcing in bladder, had to cross her legs to keep urine back. When she separated her legs the urine gushed forth.

We have in the proving of Kali-bichrom. this curious combination of symptoms, viz.: the gastric symptoms are relieved after eating, but the rheumatic symptoms appear in their place; and when the gastric symptoms reach a certain height the rheumatic pains disappear for the time being.

Never forget Kali-bich. in eating or corroding ulcers which go straight down through the tissues as if they had been bored out.

If, in a case of whooping-cough, the face of the child becomes intensely blue when coughing; stiffens itself out; frequently runs to open window for air; twitches its lower limbs in sleep, and is inclined to have screaming spells, give Ipecac, and the case will soon be well.

