# Correction

**Published:** 1891-06

**Authors:** 


Correction.—May No., page 199, third line from top should read "*cannot* bear the light of day, but *can* the artificial light at night, think of Graphites."

